# Geographic Information System-based association between the sewage network, geographical location of intermediate hosts, and autochthonous cases for the estimation of risk areas of schistosomiasis infection in Ourinhos, São Paulo, Brazil

**DOI:** 10.1590/0037-8682-0851-2020

**Published:** 2021-04-12

**Authors:** Raquel Gardini Sanches Palasio, Aline Nazaré Bortoleto, Roseli Tuan, Francisco Chiaravalloti-Neto

**Affiliations:** 1 Universidade de São Paulo, Faculdade de Saúde Pública, Departamento de Epidemiologia, Laboratório de Análise Espacial em Saúde, São Paulo, SP, Brasil.; 2 Superintendência de Controle de Endemias, Laboratório de Bioquímica e Biologia Molecular, Luz, SP, Brasil.

**Keywords:** Schistosomiasis, Biomphalaria, Spatial analysis, Gi statistics, Georeferencing, Epidemiology

## Abstract

**INTRODUCTION:**

Ourinhos is a municipality located between the Pardo and Paranapanema rivers, and it has been characterized by the endemic transmission of schistosomiasis since 1952. We used geospatial analysis to identify areas prone to human schistosomiasis infections in Ourinhos. We studied the association between the sewage network, co-occurrence of *Biomphalaria* snails (identified as intermediate hosts [IHs] of *Schistosoma mansoni*), and autochthonous cases.

**METHODS:**

Gi spatial statistics, Ripley’s K12-function, and kernel density estimation were used to evaluate the association between schistosomiasis data reported during 2007-2016 and the occurrence of IHs during 2015-2017. These data were superimposed on the municipality sewage network data.

**RESULTS:**

We used 20 points with reported IH; they were colonized predominantly by *Biomphalaria glabrata*, followed by *B. tenagophila* and *B. straminea*. Based on Gi statistics, a significant cluster of autochthonous cases was superimposed on the Christoni and Água da Veada water bodies, with distances of approximately 300 m and 2200 m from the points where *B. glabrata* and *B. straminea* were present, respectively.

**CONCLUSIONS:**

The residence geographical location of autochthonous cases allied with the spatial analysis of IHs and the coverage of the sewage network provide important information for the detection of human-infection areas. Our results demonstrated that the tools used for direct surveillance, control, and elimination of schistosomiasis are appropriate.

## INTRODUCTION

Schistosomiasis is a parasitic infection that is considered a neglected tropical disease^1^. Schistosomiasis mansoni infection in Brazil is associated with the development of the parasite *Schistosoma mansoni* Sambon, 1907 in three species of snails of the genus *Biomphalaria* (Preston, 1910), namely, *B. glabrata* (Say, 1818)*, B. tenagophila* (Orbigny, 1835)*,* and *B. straminea* (Dunker, 1848)^2^. Human infections are highly prevalent, mainly in the northeast of the country and in the southeast, where it is endemic in some areas^3^. 

In the state of São Paulo, human infections occur in specific areas where schistosomiasis endemicity is low^3^. Among these areas, the Middle Paranapanema region, where it borders with the state of Paraná, is usually reported as an important endemic region^4^. However, a recent study using spatial analysis tools in an area considered a GeoSentinel surveillance site for schistosomiasis pointed out that human schistosomiasis infections are more likely to occur in Ourinhos than in the other regions across the 25 municipalities of the Hydrographic Unit Water of Resources Management of Middle Paranapanema (UGRHI-17)^5^ ^,^ ^6^ ^,^ ^7^. Currently, Ourinhos accounts for 93% of all autochthonous cases in Middle Paranapanema^5^, with cases reported since 1952^8^ ^,^ ^9^.

The schistosomiasis cases observed in Ourinhos are probably associated with *B. glabrata*, which is a natural host to *S. mansoni* in the municipality^10^ ^,^ ^11^. This species was initially identified in Ourinhos in 1919^12^ and continues to proliferate in water bodies in this municipality^13^ ^,^ ^14^. *B. tenagophila* and *B. straminea,* two other *S. mansoni* intermediate-host (IH) species, have also been described/observed in the area^13^ ^,^ ^14^.

The spatial association between the occurrence of autochthonous cases and the presence of IHs can be analyzed using Gi spatial statistics. This tool, with the support of geographic information systems (GIS), uses the geographic coordinates of locations to find spatial clusters of a certain measure or quantity around a specific point and infer the distances at which these clusters are statistically significant^15^. Previous studies have used this tool (Gi or Gi* statistics) to analyze schistosomiasis in Africa^16^ ^,^ ^17^ ^,^ ^18^ ^,^ ^19^and vector-borne diseases, such as dengue*,* in Brazil^20^. Additionally, other studies have investigated schistosomiasis using GIS worldwide^5^ ^,^ ^21^ ^,^ ^22^ ^,^ ^23^. Thus, GIS and spatial analysis tools may contribute to identifying areas with the highest risk of human schistosomiasis infection and other diseases and consequently help guide public health measures^21^ ^,^ ^24^.

The present study used a GIS-based approach to identify rural and urban areas at risk of schistosomiasis transmission in Ourinhos (São Paulo, Brazil), combining data sources related to the presence of snails that act as *S. mansoni* IHs*,* the historical occurrence of human infection, and the sewage network.

## METHODS

### Study area

**FIGURE 1:**
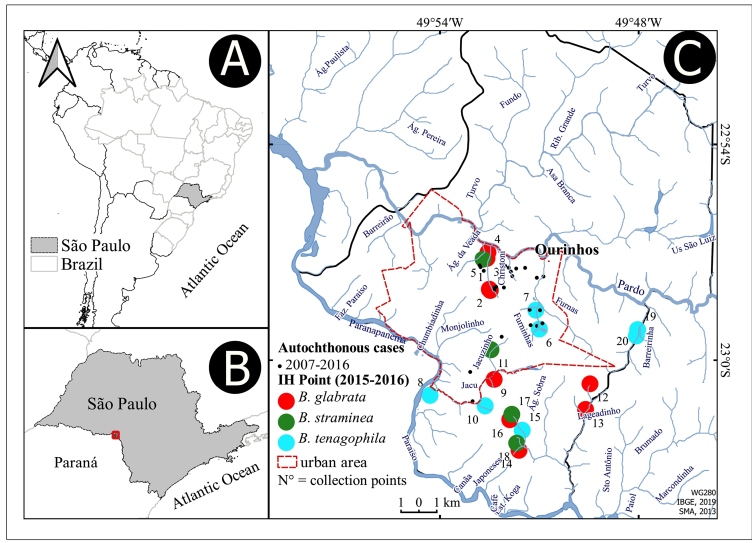
Maps of **(A)** Brazil, South America; **(B)** the state of São Paulo; and **(C)** the municipality of Ourinhos, showing the distribution of *S. mansoni* intermediate-host (IH) species (*Biomphalaria*) identified during 2015-2017, the autochthonous cases of 2007-2016, and the water bodies in Ourinhos. The numbers (N°) in Figure 1C correspond to the collection points presented in Table 1. Source: IBGE^36^ ^,^ ^37^; SMA^38^; SUCEN/Palasio et al.^14^; SINAN/CVE.

The study was conducted in the municipality of Ourinhos, southwest of the state of São Paulo (22° 58 44″ S, 49° 52 15″ W, Figure 1). The municipality extends over an area of 296 km² and had an estimated population of 113,542 inhabitants in 2019^25^ ^,^ ^26^, 97% of which lived in urban areas^25^. The municipality is covered by a variety of freshwater bodies located between the Pardo and Paranapanema rivers, which are tributaries of the Paraná river^27^.

### Data source

The geographic coordinates related to each taxonomic group identified from 20 collection points in eight of the freshwater bodies positive for IH *Biomphalaria* species and the frequency of specimens per species are displayed in Table 1. These data integrate a survey conducted in 2015-2017 at 141 sampling points located along 26 water bodies in urban, peri-urban, and rural areas in the geographical limits of the Ourinhos municipality**,** according to malacological and geospatial approaches described by Palasio et al.^14^. Of the 141 points sampled, 121 were negative for the presence of IHs or were colonized by species of *Biomphalaria* that were naturally refractory to *S. mansoni* ^14^. As reported in our preliminary and pivotal study^14^, the snails sampled were examined in the laboratory to analyze the presence of cercariae from trematodes^28^, and the species were concurrently identified through morphological characters according to Paraense (1975, 1981)^29^ ^,^ ^30^ and the DNA barcode protocol^31^ ^,^ ^32^ ^,^ ^33^. A detailed explanation of the parasitological approach and the morphological and molecular identification of snails used in this study has been provided by Palasio et al.^14^.

**TABLE 1:** Geographic coordinates and number of *S. mansoni* intermediate-host specimens of each *Biomphalaria* species collected in water bodies and points and percentage of residents served by a sewage network, septic tank, or rudimentary tank according to the census tracts of the location at the sample points in the municipality of Ourinhos, SP, Brazil, during 2015-2017.

Water body	Point**	Latitude (°)	Longitude (°)	No. of snails								Collection date	% Residents served by Census tracts*			
				*Btt*	*Bgl*	*Bst*	*Boc/Bgl*	*Boc/Btt*	*Boc/Bst*	*Boc*	*Bsp*		*sewage network*	*tank*		Other ***
														*sep*	*rud*	
Christoni	1	-22.967600	-49.874683	-	170	-	-	-	-	-	42	2015-2016	89.3	-	10.3	0.3
	2	-22.967117	-49.875167	-	25	-	-	-	-	-	21	2015-2017				
	3	-22.952833	-49.876333	-	45	-	68	-	-	-	34	2015-2016	95.6	0.6	3.7	-
	4	-22.950050	-49.875850	-	25	-	10	-	-	14	28	2015-2016				
**Água da Veada**	5	-22.953222	-49.878306	-	-	51	-	-	-	59	104	2015-2017	95.6	0.6	3.7	-
**Furninhas**	6	-22.985556	-49.849972	125	-	-	-	-	-	50	-	2015	7.8	86.3	5.9	-
	7	-22.976766	-49.851745	32	-	-	-	-	-	-	-	2015	100	-	-	-
**Jacu**	8	-23.016306	-49.905000	-	-	-	-	31	-	24	7	2015-2016	-	-	-	-
	9	-23.008944	-49.872750	-	59	-	-	-	-	-	41	2015-2017	1.8	0.7	96.1	1.1
	10	-23.021167	-49.877278	-	-	-	-	98	-	47	53	2015-2016	-	-	-	-
**Jacuzinho**	11	-22.995111	-49.874333	-	-	20	-	-	-	-	61	2015-2016	98.4	1.3	0.3	-
**Lageadinho**	12	-23.010972	-49.824500	-	12	-	-	-	-	-	-	2015	19.1	9.2	71.6	-
	13	-23.022833	-49.826733	-	31	-	-	-	-	-	-	2016				
**Sobra**	14	-23.041650	-49.860233	-	35	-	-	-	-	-	-	2016	-	-	-	-
	15	-23.032400	-49.858617	57	-	-	-	-	-	-	-	2016	-	-	-	-
	16	-23.027467	-49.864867	-	46	-	-	-	-	-	-	2016	0.9	7.1	91.9	-
	17	-23.025063	-49.863847	-	-	38	-	-	-	-	-	2016	-	-	-	-
	18	-23.038333	-49.861400	-	-	-	-	-	53	-	17	2016	-	-	-	-
**Barreirinha**	19	-22.985800	-49.800517	27	-	-	-	-	-	-	-	2016	-	5.3	94.7	-
	20	-22.988750	-49.801167	142	-	-	-	-	-	-	-	2016-2017				
**Total**				**383**	**448**	**109**	**78**	**129**	**53**	**194**	408	-	-	-	-	-

*Source: IBGE^25^, ** SUCEN/Palasio et al.^14^, numbers presented in Figure 1 ***Others = ditch, river, lake, or other sewer.

***Bgl*: *B. glabrata, Bst*: *B. straminea, Btt: B. tenagophila, Boc: B. occidentalis, Bsp: Biomphalaria*** spp., **sep:** septic, **rud:** rudimentary.

Information regarding schistosomiasis cases from 2007 to 2016, including notification date, residence geographical location, probable infection site (PIS), and epidemiologic classification, was obtained from the National Notifiable Disease Information System (SINAN). Access to the necessary information was provided by the Alexandre Vranjac Center for Epidemiologic Surveillance (CVE). We used this information to obtain the frequencies of the occurrence of autochthonous, imported, and unknown-origin cases in the municipality.

Once the residence geographical location for each autochthonous schistosomiasis case was known, the batch geocoding tool^34^, which uses Google Earth, was used to obtain the respective geographic coordinates (datum WGS84). Cartographic material (maps with rivers, census tract layers, and sanitation data) was obtained from the Brazilian Institute of Geography and Statistics (IBGE)^25^ ^,^ ^35^ ^,^ ^36^ ^,^ ^37^, the Secretariat for the Environment of the State of São Paulo (SMA)^38^, and the National Secretariat for Environmental Sanitation (SNSA) in partnership with the Brazilian National Water Agency (ANA)^39^. The points corresponding to the geographic coordinates of snail IHs and autochthonous schistosomiasis cases were imported into and viewed using QGIS software version 3.10.5^40^. 

A fundamental part of the current study was the data related to the percentages of residents served by the sewage system, septic tanks, and/or rudimentary tanks^25^, according to census tracts^35^. These data were computed using the MMQGIS plugin^41^coupled with spatial join geographic operation, both available on QGIS version 3.10.5^40^. To do this, we considered the map of the census tracts, statistical data from the 2010 census^25^ ^,^ ^35^, and geographical coordinates of IHs.

### Data analysis

The relationship between the spatial distribution of autochthonous cases of schistosomiasis from 2007 to 2016 and the presence of IHs from 2015 to 2017 was analyzed using Gi spatial statistics, which is an indicator of local spatial association^15^ ^,^ ^42^. These statistics considered the occurrence of autochthonous cases around the points where IH snails were found.

The schistosomiasis incidence rates by census tracts were calculated using the MMQGIS plugin^41^ in QGIS^40^. In the 2010 population^25^, the centroid coordinates of the census tracts^35^ and the coordinates of georeferenced schistosomiasis cases were considered. Gi statistics were calculated for each geographic coordinate where IHs were detected, taking into account the incidence rates. This allowed us to obtain a profile of schistosomiasis based on the spatial pattern of autochthonous schistosomiasis cases and its relationship with freshwater bodies and the IH snails that colonize them. A significance level of 5% was used, which corresponded to the minimum value of the Gi statistics (3.2889) (N = 100) according to Table 3 of the paper published by Ord and Getis^15^. 

The application of the Gi statistics took into account the measured attribute values in the pairs of coordinates corresponding to the locations analyzed (schistosomiasis incidence rates in census tract centroids). As it is a focal statistic, it considered the pair of geographic coordinates of each focus with IH (*i*) without taking into account the value of the attribute at this point^15^.

Gi can be written as Σ_j_w_ij_(d) x_j_ / Σ_j_x_j_, for i ≠ j, where *j* represents the geographic coordinates of the centroid of the census tracts where there are schistosomiasis cases, W_ij_ the binary and symmetric matrix that defines the neighborhood between the areas, x_j_ the values of the incidence rates of the cases in the position of each *j*, and *d* the measure of the distance established by the neighborhood model. This calculation was performed with the sum of neighboring samples in relation to the position of *i*, wherein the value *xi* was not included in the sum as the place where IHs were collected, and the incidence rate was considered null (= 0)^15^.

In the present study, a significant result for these statistics would indicate that the location in question may be considered a potential infection area for schistosomiasis. Gi statistics were calculated using the ‘spdep’ package^43^ in R version 3.2.2^44^. The presence of clusters was investigated using a maximum distance of 4000 m between each point where IHs were present and the centroids of the census tracts.

Furthermore, the spatial dependence between the distribution of points corresponding to autochthonous cases of schistosomiasis and those corresponding to places where the IHs were found was evaluated by considering the geographic coordinates of the autochthonous cases and the IH, using Ripley’s K12-function^45^ ^,^ ^46^ and the R software version 3.2.2^44^ with the ‘Splancs’ package^47^. We used the borders of the study area in a shapefile format and considered the coordinates of the cases and IHs in the UTM format. The result of the K12-function allowed us to verify the radius of influence, which is the limited and statistically significant distance where a positive spatial dependence between the two distributions of points occurs. We used the geographic coordinates of the autochthonous cases of schistosomiasis and the radius of influence data of the K12-function result to estimate the kernel density with a plugin available in the program QGIS version 3.10.5^40^.

With the coordinates of the points where IHs were found and the radius of influence of each point analyzed in the Gi statistics (distances considered significant, higher limit), it was possible to identify the clusters of autochthonous cases around points with IHs. We performed this procedure using the MMQGIS plugin^41^ and created a buffer geographic operation available on QGIS^40^. We merged all clusters into one cluster, which was restricted to the Ourinhos urban area. This cluster map obtained using Gi statistics was superimposed onto the respective autochthonous case hotspots obtained using the kernel tool.

### Ethical considerations

The project was approved by the Faculdade de Saúde Pública of Universidade de São Paulo, Committee for Ethics in Research, the Plataforma Brasil system, Ministério da Saúde - Conselho Nacional de Saúde (number, CAAE: 53559816.0.0000.5421).

## RESULTS

### IH occurrence in Ourinhos

**FIGURE 2:**
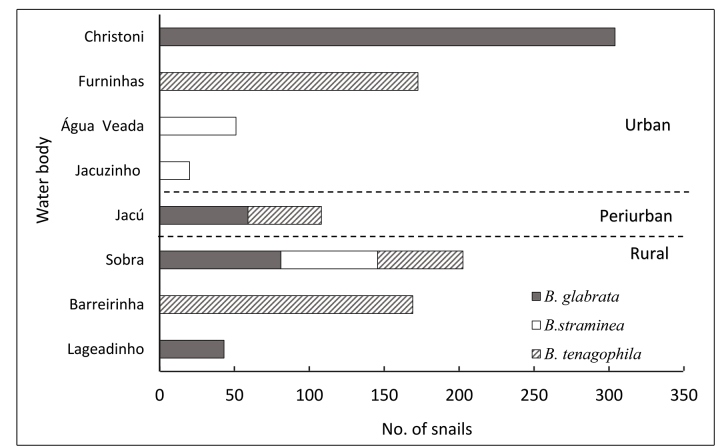
Number of intermediate-host specimens (*Biomphalaria* species) of *S. mansoni* collected during 2015-2017 in eight water bodies in the urban, peri-urban, and rural areas of the municipality of Ourinhos, SP, Brazil.

The geographical occurrence points for *B. glabrata*, *B. tenagophila*, and *B. straminea* over the 20 locations sampled across eight freshwater bodies assigned as breeding sites for IHs demonstrated the predominant occurrence of *B. glabrata* (Figure 1C)*.* We also found that the relative abundance of *B. glabrata* was higher in Christoni, Sobra, and Jacu than that of the other two IHs investigated (Table 1, Figure 2). However, none of the snails were infected with *S. mansoni* in the parasitological analysis, as reported by Palasio et al.^14^. Overall, the three natural *S. mansoni*-IH species occurred in the urban, peri-urban, and rural areas of Ourinhos (Figure 1C, Figure 2).

### Frequency of schistosomiasis and PIS and their relationship with the IH and sewage system

**FIGURE 3:**
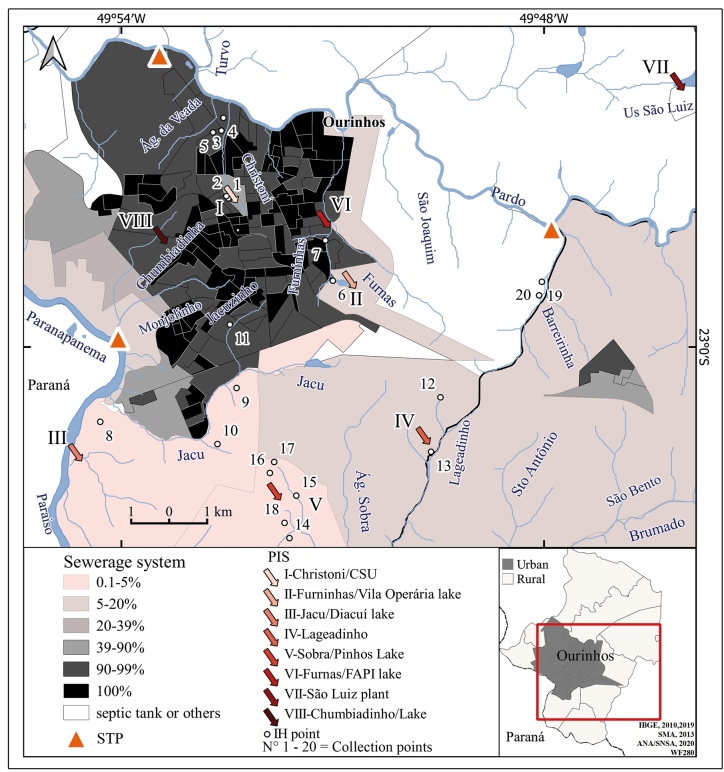
Map of the municipality of Ourinhos, state of São Paulo, Brazil, highlighting the main probable infection site (PIS), percentage of residents served by a sewage network according to the census tracts, sewage treatment plants (STPs), and points where *S. mansoni* intermediate hosts (IHs) were found. The numbers (N°) in this figure correspond to the collection points presented in Table 1. Source: IBGE^25^ ^,^ ^35^ ^,^ ^36^ ^,^ ^37^; SMA^38^; ANA/SNSA^39^.

The frequencies of autochthonous, imported, and unknown-origin cases were 39.7% (25), 7.9% (5), and 52.4% (33), respectively. On average, 6.3 cases per year were detected. Using information from the PIS in the epidemiological survey records, eight PIS were geocoded in the water bodies of Christoni, Furninhas, Jacu, Sobra (Pinhos Lake), Lageadinho, Chumbiadinha (Lake), Furnas (Fapi Lake), and Lake of São Luiz plant (Figure 3). It is noteworthy that the presence of IHs was only found in the first five locations, the majority of PIS were vague, and information was not available for 48% of them, making geocoding impossible.

In the water bodies of Chumbiadinha, Lake of São Luiz plant*,* and Furnas, *B. glabrata* were reported until 2009, 2009, and 2012, respectively^13^ ^,^ ^48^ ^,^ ^49^. Currently, only *B. occidentalis* Paraense, 1981 has been identified in these sites^14^, and this species is not susceptible to *S. mansoni* ^50^. IHs were found in overlapping areas with a high percentage of residents served by the sewage network as well as in places served by tanks or other systems (Figure 3, Table 1). 

### Association between IH occurrence and autochthonous cases

**FIGURE 4:**
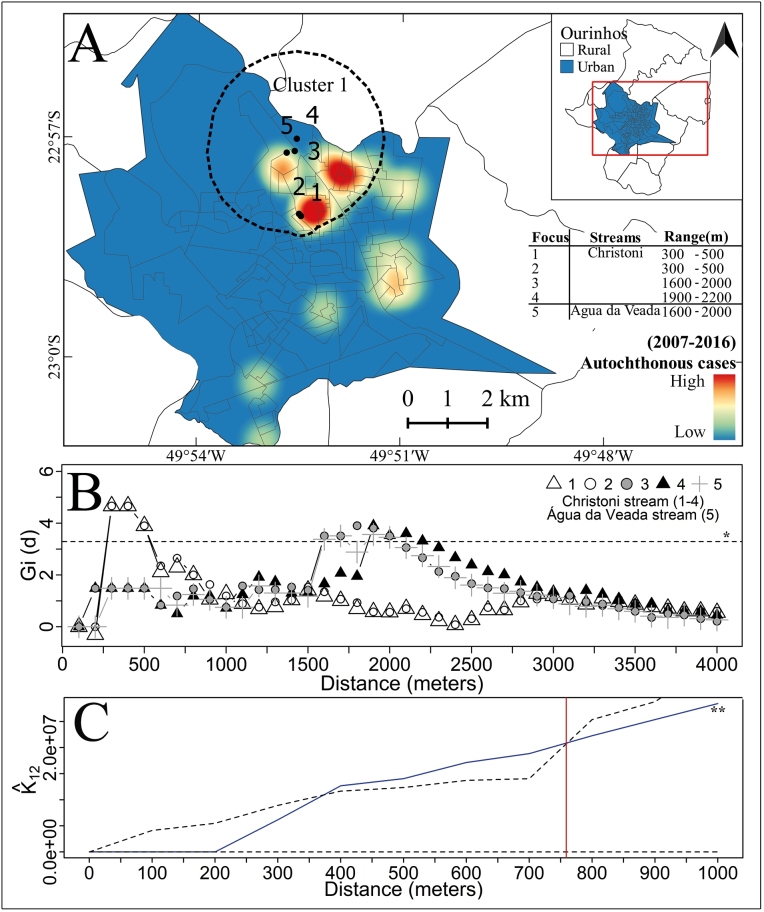
**(A)** Kernel density map of the urban area (759 m radius of influence) showing the distribution of autochthonous schistosomiasis cases and significant clusters in the Gi statistics of cases around sampling points with intermediate hosts (foci). **(B)** Graph showing significant clusters of autochthonous cases around the Christoni stream and Água da Veada stream sampling points with IH. **(C)** Graph of the bivariate K12-function analysis in Ourinhos, SP, Brazil, during the 2007-2016 period. (B)* Statistically significant values are above the horizontal line (G*i* [d] > 3.28, *P* < 0.05); (C)** The blue curve above the envelope shows a positive spatial dependence between the autochthonous cases of schistosomiasis and the IH up to a distance of ~759 m.

Figure 4A shows the results of the Gi statistics with locations considered potential risk areas for human schistosomiasis infection as well as the extent of the concatenated clusters of autochthonous cases around these points and the kernel density map.

Significant clusters of autochthonous cases were superimposed on the Christoni stream region from approximately 300 m (lower limit) to 2200 m (higher limit) from sites where *B. glabrata* was detected. Another cluster was superimposed on the Água da Veada stream region at a distance of approximately 1600-2000 m (Figure 4A-B) from the *B. straminea* collection site^14^ (Table 1). All clusters were combined into a single cluster (Cluster 1 in Figure 4A). 

The graph obtained using the K12-function shown in Figure 4C indicates a positive spatial dependence up to a distance of approximately 759 m between autochthonous cases and IH snails.

## DISCUSSION

In this study, the association of data from autochthonous cases reported in Ourinhos (2007-2016) with the spatial location of IHs and the sewage network allowed the identification of the Christoni freshwater body as the most suitable area for human schistosomiasis infection. The results obtained through statistics corroborated previous results^5^ ^,^ ^51^_,_ which showed that the Christoni stream was the area most probably at highest risk of peridomestic schistosomiasis transmission^51^ ^,^ ^52^. Interestingly, in addition to the identification of specific points, the results obtained using Gi statistics provided important information regarding the significant distances that should relate to the local occurrence of schistosomiasis infection, a phenomenon that has not been previously reported in the literature.

The inventory of species in the Christoni stream shows that *B. glabrata* is abundant and predominates at four specific points^14^, which corroborates data from previous surveys^10^ ^,^ ^13^ ^,^ ^48^ ^,^ ^49^. Although the *B. glabrata* samples collected were reported to be negative for cercariae infection, this species is known to be the most suitable IH for the development and transmission of the parasite^11^ ^,^ ^53^. Based on previous studies, it is considered the most competent IH species for *S. mansoni* transmission in the Paranapanema region^51^ ^,^ ^54^.

In Ourinhos, *B. glabrata* predominance in census tracts, where sanitary sewage is still performed using a septic and/or rudimentary tank, is relevant and of concern from a medical perspective. In the case of the census tracts where the Christoni stream is located, 89.3% of the residents are served by a sewage network and 10.6% by a rudimentary tank and other drains^25^. This percentage pattern of sanitary sewage is a potential explanation for the decrease in the number of autochthonous cases in recent years as well as for the maintenance of focal transmission in this municipality^5^.

*B. straminea* is resilient to extreme environmental variations and is capable of adapting to altered environments^55^. The presence of the *B. straminea* IH in the Água da Veada stream, where previous surveys indicated colonization by *B. tenagophila* ^13^ and *B. glabrata* ^49^ ^,^ ^56^, is further evidence of the expansion potential of *B. straminea*. Regarding the natural susceptibility of *B. straminea* to *S. mansoni*, the physiological adaptation of this species to the parasite is relatively high in snails inhabiting regions of northeastern Brazil^57^. The occurrence of *B. straminea* in Água da Veada and the fact that a portion of the residents living nearby are still served by septic (0.6%) or rudimentary (3.7%) tanks^25^ suggest the need for enhanced surveillance of areas colonized by this species. Although designated as a statistically significant cluster for schistosomiasis infection, in Água da Veada are more likely to result from a bias associated with its closeness to Christoni. 

Although the Gi statistics considered only Christoni to have significant associations, the water bodies of Sobra, Lageadinho, and Jacu require monitoring, as *B. glabrata* ^14^ is also present therein. In addition, the percentage of residents served by the sewage system near these areas was below 20%^25^. *B. glabrata* presence has been registered in the water bodies of Jacu, located in peri-urban areas, in the past and continues today^13^ ^,^ ^14^ ^,^ ^58^. In the Sobra and Lageadinho water bodies, located in rural areas, the first record of this species was in 2009^49^. Until then, only *B. straminea* had been recorded in Sobra water bodies^59^.

The results of this study demonstrate that the use of GIS tools in association with malacology, epidemiological data, and sewerage infrastructure has the potential to improve schistosomiasis control, fostering the use of new technologies to locally eliminate future infections.

One of the limitations of this study is the discrepancy between the periods of collection of snails (2015-2017) and of data on schistosomiasis cases (2007-2016). Despite this incoherence, such a limitation may be overcome by comparing our results with those from the literature^13^ ^,^ ^48^ ^,^ ^49^ ^,^ ^54^ ^,^ ^56^ ^,^ ^58^ ^,^ ^59^.

In the 2007-2016 period, the Gi statistics made it possible to exclusively identify the Christoni stream as a location characterized by significant clusters of autochthonous cases associated with the presence of *B. glabrata*. Therefore, this species is a candidate for the main target of environmental monitoring measures in this municipality. In addition, the use of this technique allowed us to verify that the association between the residence geographical location of autochthonous cases and the spatial distribution of IH provides vital information regarding potential transmission areas. Despite the absence of cercariae in the samples of *B. glabrata* collected in Ourinhos, the high susceptibility of this species to *S. mansoni* in laboratory conditions^11^ ^,^ ^60^ indicates the risk of schistosomiasis persistence in this region.

Moreover, the Gi statistics partially overcame the limitation related to the lack of precise information regarding the location of the PIS, which is consistent with the information that characterizes the transmission of schistosomiasis as being predominantly peridomestic^51^. The proximity between water bodies and residences is another typical characteristic of the incipient urbanization process.

The fact that the significant distance in the Gi statistics is approximately 2 km allowed us to calibrate the surveillance activities to a concise and statistically pre-established area. Accordingly, it is possible to develop schistosomiasis control and monitoring activities at well-defined focal points, rationalizing the use of public resources, since Brazil spent approximately 7.7 million dollars in 2015 to control the infection^61^. Therefore, the information presented in this study as well as the tools used may be adequate to develop and direct surveillance actions that contribute to the control and even elimination of schistosomiasis in the municipality of Ourinhos.
